# Homologous *Escherichia coli* Identified in Cerebrospinal Fluid and Bloodstream

**DOI:** 10.3389/fcimb.2021.674235

**Published:** 2021-09-10

**Authors:** Qingmiao Shi, Jun Zhang, Jinghui Wang, Lijuan Du, Zhaoyang Shi, Min Xu, Yonggang Luo

**Affiliations:** ^1^Gene Hospital of Henan Province, The First Affiliated Hospital of Zhengzhou University, Zhengzhou, China; ^2^Department of Infectious Diseases, The First Affiliated Hospital of Zhengzhou University, Zhengzhou, China; ^3^Department of Pharmacy, The First Affiliated Hospital of Zhengzhou University, Zhengzhou, China; ^4^Department of Intensive Care Unit, The First Affiliated Hospital of Zhengzhou University, Zhengzhou, China; ^5^Department of Clinical Laboratory, The First Affiliated Hospital of Zhengzhou University, Zhengzhou, China

**Keywords:** *Escherichia coli*, whole genome sequencing, phylogenetic analysis, homology, craniopharyngioma, metagenomic next-generation sequencing

## Abstract

**Background:**

*Escherichia coli* is an opportunistic bacterium that causes a wide range of diseases, such as bloodstream infection and central nervous system infection. The traditional culture-based method to detect *E. coli* usually takes more than 2 days. The object of this study is to explore the value of metagenomic next-generation sequencing (mNGS) in identifying *E. coli* from human cerebrospinal fluid. In addition, we investigated the infection source of *E. coli* through whole genome sequencing and phylogenetic analysis.

**Methods:**

We combined a clinical example to analyze the function of mNGS in pathogen detection from cerebrospinal fluid. NextSeq 550Dx platform was applied for mNGS. Next, whole genome sequencing was performed to obtain the genomic characterization of *E. coli*. Furthermore, we screened 20 *E. coli* strains from the National Center for Biotechnology Information and conducted a phylogenetic analysis.

**Results:**

A middle-aged patient who attended our hospital was diagnosed with craniopharyngioma and received surgery. The patient had recurrent fever and persistent lethargy after surgery. Cerebrospinal fluid culture firstly failed to grow the bacteria. Next the cerebrospinal fluid sample was detected by mNGS and the sequence readings of *E. coli* were identified. Later, *E. coli* was reported *via* the second cerebrospinal fluid culture, certifying the result of mNGS. Moreover, we also cultured carbapenem-resistant *E. coli* from the patient’s bloodstream. Through whole genome sequencing and phylogenetic analysis, we found that the *E. coli* isolated from cerebrospinal fluid and the bloodstream was 100% homologous, indicating the *E. coli* central nervous system infection was originated from the bloodstream.

**Conclusion:**

Metagenomic next-generation sequencing is a valuable tool to identify the pathogens from cerebrospinal fluid, and seeking the infection source is of great significance in clinical diagnosis and treatment. Furthermore, carbapenem-resistant *E. coli* is a serious problem as the cause of bloodstream infection and central nervous system infection, and effective and adequate measures to prevent and control the present circumstance are urgent.

## Introduction

*Escherichia coli* is a common gram-negative bacterium that can be a harmless inhabitant of the gastrointestinal tract in humans (Croxen et al., 2013). These symbiotic *E. coli* strains seldom contribute to illness. However, once the individual’s immune dysfunction or gut barrier is destroyed, it is also pathogenic and can result in many diseases, including diarrheal and extraintestinal diseases such as bloodstream infection, urinary tract infection, and central nervous system (CNS) infection (Kaper et al., 2004). The traditional diagnosis for pathogenic *E. coli* has depended on culture-based techniques (Peña-Gonzalez et al., 2019), but it usually takes more than 2 days. With the development of molecular diagnostic tools such as metagenomic next-generation sequencing (mNGS), it provides a handy sensitivity method to rapidly detect the pathogens (Li et al., 2021).

Craniopharyngioma is a rare benign epithelial tumor of the CNS derived from residual cells of Rathke’s pouch, which includes two different histopathological type, adamantinomatous and papillary craniopharyngioma (Müller, 2014). It constitutes about 2–5% of primary intracranial tumors with an overall morbidity of 0.5–2 cases per million (Erfurth et al., 2013). Approximately 60% of all cases emerge in children, and the second peak of onset is the age of 40 to 44 years old. The invasion, adhesion, and compression of key structures around the sellar region lead to high mortality of craniopharyngioma even while receiving treatment (Karavitaki et al., 2005; Zoicas and Schöfl, 2012). The most common postoperative complications are involved in neuroendocrine dysfunction, visual deficits, and hypothalamic metabolic disorder. Although intracranial infection is seldom reported after surgery, it’s a serious problem and shouldn’t be ignored by clinicians.

**Figure 1:**
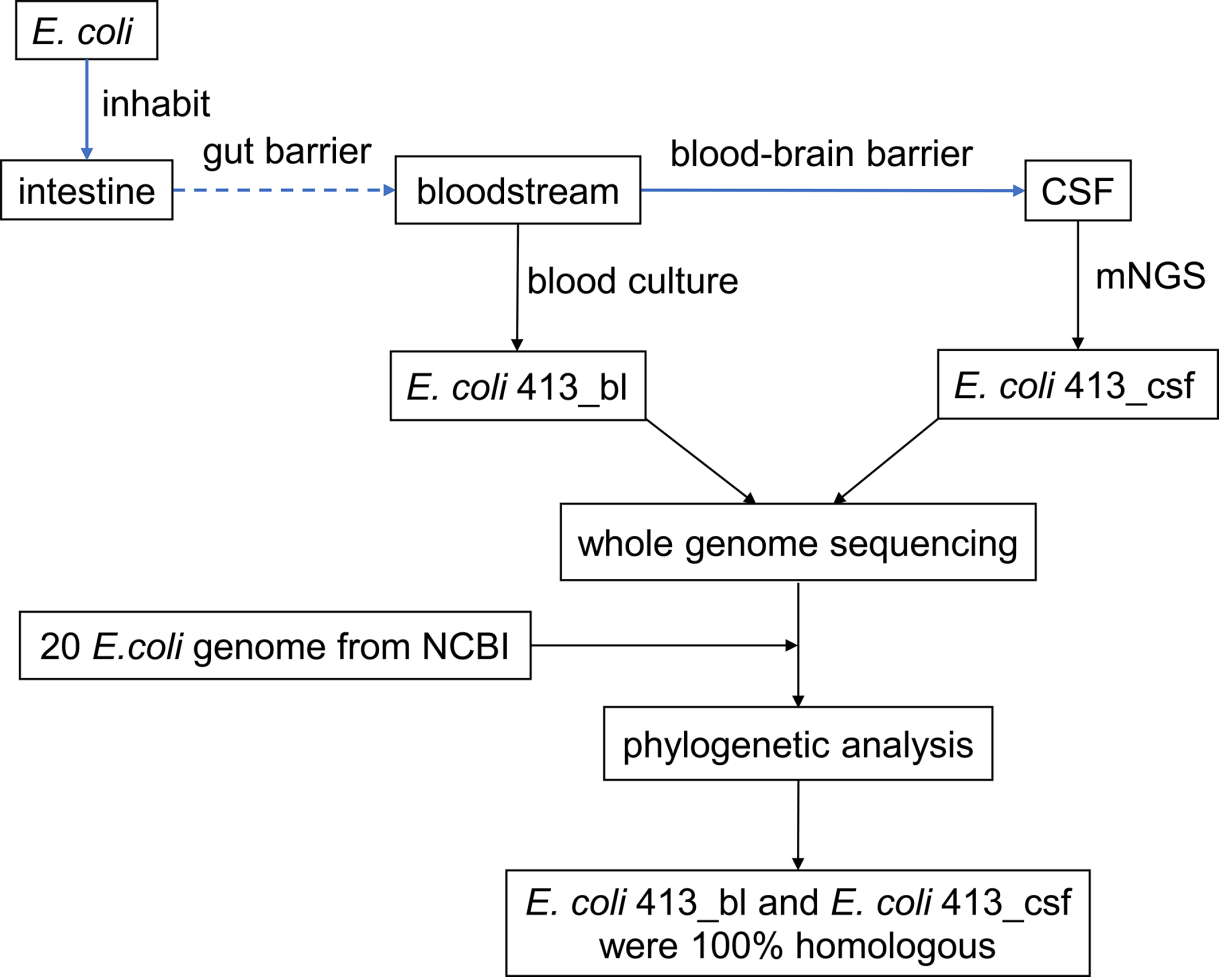
Study design and flow diagram. CSF, cerebrospinal fluid; mNGS, metagenomic next-generation sequencing; NCBI, National Center for Biotechnology Information.

Our study herein reported a febrile and lethargic patient who had undergone excision surgery of adamantinomatous craniopharyngioma. We identified *E. coli* sequence readings from the patient’s cerebrospinal fluid (CSF) by mNGS when the traditional CSF culture first failed to grow a pathogen. Furthermore, we confirmed that the *E. coli* intracranial infection was sourced from bloodstream through whole genome sequencing and phylogenetic analysis, thus providing a modest recommendation for clinical management **(** **Figure 1** **)**.

## Materials and Methods

### Patient and Samples

Our study involved a middle-aged patient who was diagnosed with craniopharyngioma and received surgery at our hospital. Because of recurrent fever and persistent lethargy after surgery, the patient’s CSF sample was collected after lumbar puncture by an experienced intensive care unit doctor, and the *E. coli* sequence readings were detected from CSF by mNGS. In addition, two suits of blood cultures were taken for the patient; one suit was from peripheral vein and another from central venous line. Each set included an aerobic bottle and an anaerobic bottle. And all the four bottles cultured carbapenem-resistant *E. coli*. The antimicrobial susceptibility test was detected by Kirby-Bauer and broth dilution method.

### Metagenomic Next-Generation Sequencing

After CSF sample preprocessing, the microbial genomic DNA extraction was performed by TIANamp Micro DNA Kit (TIANGEN Biotech, Beijing) according to the manufacturer’s instruction. Next, library construction was carried out, including genomic DNA fragmented, end-repair, 3’ adenylated, adapters ligation, PCR amplifying, and purified products. The qualified single-stranded circular DNA library was sequenced on NextSeq 550Dx platform (illumina, San Diego, USA) with a single-end 75bp sequencing strategy. We obtained the sequencing depth (**Supplementary 1**) using the complete genome of *Escherichia coli* str. K-12 substr. MG1655 (NCBI Reference Sequence: NC_000913.3) as genome reference sequence. Finally, we conducted a bioinformatics analysis and determined the mNGS-based pathogenic microorganism. The result revealed that mNGS identified 32,296 sequence readings of *E. coli* in the CSF sample.

### Whole Genome Sequencing

Next, the *E. coli* strains isolated from CSF and the bloodstream were sequenced by whole genome sequencing, respectively. Firstly, the genomic DNA of *E. coli* was extracted by using MagPure DNA Kit (Cat. No. MD5118-05F, Magen, China). Secondly, we determined the concentration and purity of DNA through Qubit fluorometer and Nanodrop 2000 spectrophotometer (Thermo Fisher Scientific, Carlsbad, CA, USA). Thirdly, library construction and whole genome sequencing was performed on the MGISEQ-2000 platform at BGI (Shenzhen, China). The antibiotic-resistant genes were annotated using CARD (Comprehensive Antibiotic Resistance Database Version 3.1.2).

### Screening of *E. coli* Strains

Twenty *E. coli* strains with genome information were screened out from the National Center for Biotechnology Information (https://www.ncbi.nlm.nih.gov). The 20 *E. coli* strains meet the following criteria: (1) the hosts were homo sapiens or human; (2) the isolation source was blood, CSF, or feces; (3) the collection date was in the last 10 years, except that the strain CE10 isolated from CSF was collected in the 1970s. The genome assembly and annotation data of the 20 *E. coli* strains were downloaded from Genbank (https://ftp.ncbi.nlm.nih.gov/genomes/genbank/) for a genomic phylogenetic analysis.

### Phylogenetic Analysis

Roary, a pipeline that structures large-scale pan genomes at a high speed (Page et al., 2015), was used to construct the pan genome and core genome of 22 *E. coli* strains. Next, we adopted ClonalFrameML to analyze and detect recombination in bacterial whole genomes (Didelot and Wilson, 2015). Single nucleotide polymorphisms distance matrix with snp-dists was calculated. Furthermore, we performed phylogenetic analysis and a phylogenetic tree of 22 *E. coli* strains with branch lengths was reconstructed to display the evolutionary relationships.

## Results

### Case Presentation

**Figure 2:**
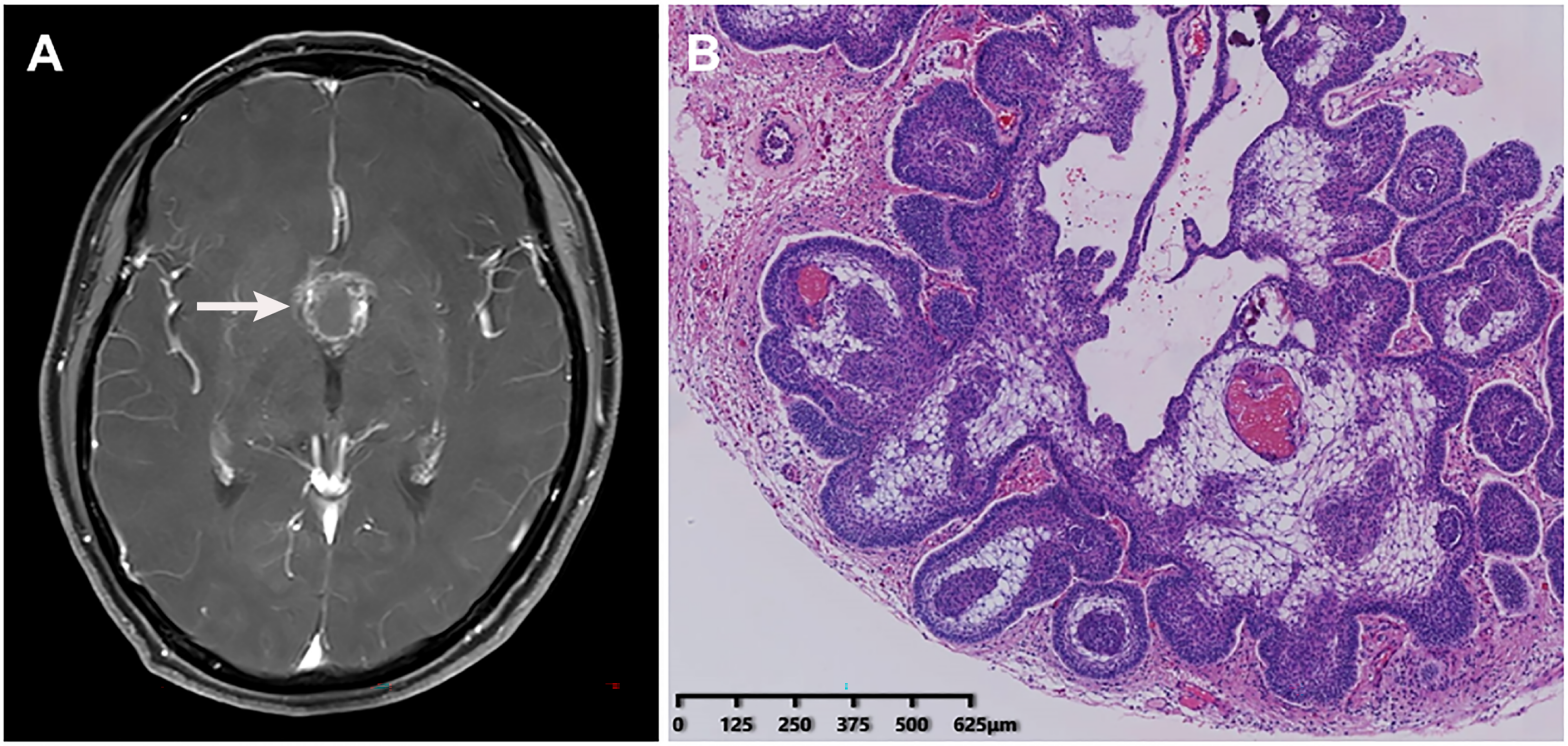
Radiological imaging and histopathological examination of the patient. **(A)** Head magnetic resonance imaging showed a suprasellar space-occupying lesion. **(B)** Histopathological examination of a surgical specimen indicated adamantinomatous craniopharyngioma.

A middle-aged patient who complained of blurred vision with headache for three years was admitted to the neurosurgery department on July 23, 2020. The patient had a two-year history of type II diabetes and received oral hypoglycemic drugs regularly. Laboratory tests showed the fasting plasma glucose of 6.76 mmol/L, with glycated hemoglobin of 7.10%. Other tests were within normal range including blood routine examination, blood biochemistry, coagulation function, and thyroid function. Head magnetic resonance imaging scanning indicated a suprasellar space-occupying lesion **(** **Figure 2A** **)**, which was suspected as craniopharyngioma. After a thorough evaluation, on August 3, the patient was administered the resection of the lesion and repair of CSF rhinorrhea by endoscopic endonasal transsphenoidal approach, which was confirmed to be adamantinomatous craniopharyngioma by histopathological examination of a surgical specimen afterwards **(** **Figure 2B** **)**.

Despite using cefotiam (1g, q12h) as prophylactic treatment for surgery, the patient developed fever and lethargy the following day post-surgery. Reexamination of blood routine reported raised white blood cell of 13.49×10^9^/L (reference range 3.9–9.5×10^9^/L) and percentage of neutrophils of 92.1% (reference range 40–75%). The hemoglobin content 106.0 g/L (reference range 115–150 g/L) and percentage of lymphocyte 6.2% (reference range 20–50%) were reduced. Lumbar puncture was performed and CSF tests revealed elevated glucose 15.29 mmol/L (reference range 2.5–4.4 mmol/L), total protein 5982.00 mg/L (reference range 100–300 mg/L), and count of white blood cells 192.00×10^6^/L. Continuous monitoring of the CSF indicated no sign of CNS infection. Bacterial culture and gram stains of CSF reported negative results, respectively. The patient was given empiric anti-infective treatment with latamoxef sodium (1.5g, q12h) and vancomycin (1g, q12h) intravenously. Six days later, the patient’s condition was improved.

On August 18, the patient occurred CSF rhinorrhea. Repair surgery of CSF rhinorrhea was performed by endoscopic endonasal transsphenoidal approach immediately. However, the patient developed fever and lethargy again the day after surgery, which had high possibility of intracranial infection considering that the characteristics of CSF was bloody and cloudy. Vancomycin (1g, q12h) and cefoperazone-sulbactam (3g, q12h) were given for anti-infection intravenously, but the fever didn’t reduce. To make matters worse, the patient emerged in diabetic ketoacidosis and was transmitted to intensive care unit urgently. CSF was bloody and cloudy with 0.77 mmol/L of glucose, and the glucose ratio was 0.06. The count of white blood cells in CSF was increased to 187385.00×10^6^/L and the total protein was 4098.5 mg/L. But the bacterial culture of CSF was negative as before. In order to further seek the pathogen, the CSF was tested by mNGS and 32,296 sequence readings of *E. coli* was identified in CSF. Next, *E. coli* was also reported *via* the second cerebrospinal fluid culture, certifying the result of mNGS. After consultation with the pharmacy department, meropenem (2g, q8h), tigecycline (100mg, q12h), and linezolid (0.6g, q12h) were applied intravenously for this patient. To our surprise, carbapenem-resistant *E. coli* was cultured in the patient’s bloodstream later. The minimum inhibitory concentration (MIC) was measured by broth dilution method. The antimicrobial susceptibility test showed a multi-drug resistance of *E. coli* to doxycycline (MIC ≥ 16 ug/ml), piperacillin-tazobactam (MIC ≥ 128 ug/ml), cefoperazone/sulbactam (MIC ≥ 64 ug/ml), ceftazidime (MIC ≥ 64 ug/ml), cefepime (MIC ≥ 32 ug/ml), aztreonam (MIC ≥ 64 ug/ml), meropenem (MIC ≥ 16 ug/ml), imipenem (MIC ≥ 16 ug/ml), levofloxacin (MIC ≥ 8 ug/ml), and so on.

Questioning the medical history of the patient, we came to know that the patient had symptoms of diarrhea after the surgery for craniopharyngioma. Thus, we inferred that the gut microbiota translocations occurred in the state of surgical stress. The *E. coli* residing in the intestine became opportunistic bacterium and translocated into the blood, resulting in bloodstream infection. Furthermore, the *E. coli* in the bloodstream moved through the blood-brain barrier and entered the CSF, causing CNS infection. To prove the conjecture, whole genome sequencing analysis of *E. coli* strains isolated from CSF sample and bloodstream sample were performed. After phylogenetic analysis, the results suggested that the *E. coli* from CSF was 100% homologous with the *E. coli* from the blood. It indicated that the intracranial *E. coli* infection of the patient was not caused by the invasive surgical procedure but originated from the bloodstream. So far, the cause of the patient’s intracranial infection can be clarified.

**Figure 3:**
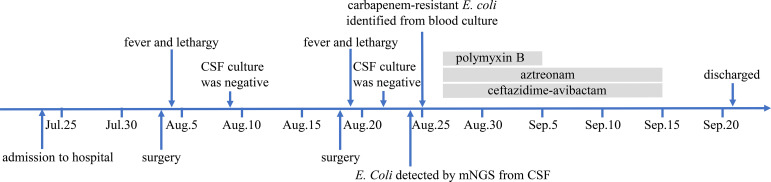
The schematic of the patient’s clinical course. CSF, cerebrospinal fluid; mNGS, metagenomic next-generation sequencing.

According to combined drug susceptibility test, the anti-infective regimen was adjusted to intravenous administration of ceftazidime-avibactam (2.5g, q8h, ivgtt) and aztreonam (2g, q6h, ivgtt), along with intrathecal injection of polymyxin B (5WIU). Considering the neurotoxicity of polymyxin B, its use was discontinued after seven times intrathecal injections. Through 20 days of anti-infection treatment, the fever of this patient was controlled and her condition was getting better. CSF was transparent with white blood cells count of 53×10^6^/L, glucose of 5.49 mmol/L, and total protein of 1319.0 mg/L. Next the antibiotics were discontinued, the patient remained in the hospital under observation for a week, and then was discharged. Two weeks later, we followed up with the family of the patient. Unfortunately, we learned that the patient has expired outside hospital due to the uncontrolled infection caused by newly developed and untreated CSF leak. The schematic of the patient’s clinical course was showed in **Figure 3**.

### The *E. coli* Strains Identified in Our Study

From the CSF sample, we detected 32,296 sequence readings of *E. coli* genome by mNGS and this strain *E. coli* was named 413_csf. From the blood culture sample, we identified carbapenem-resistant *E. coli*, which was named 413_bl. Through whole genome sequencing, it showed that the *E. coli* 413_bl and 413_csf were homologous. The antibiotics-resistant genes data of whole genome sequencing was presented in **Supplementary 2**. In addition, the carbapenem resistance arose from β-lactam ring hydrolysis by dedicated carbapenems enzymes and the carbapenems come from ambler class B metallo-β-lactamases.

### Genomic Characteristics

We filtered out 20 *E. coli* strains with whole genome sequencing information from the National Center for Biotechnology Information. The genomic characteristics of 20 *E. coli* strains were presented in **Table 1**. Fourteen *E. coli* strains were isolated from the blood of homo sapiens; one strain was from CSF and five strains were from feces. The genome size and protein-coding sequences for 20 *E. coli* strains ranged from 4.82 Mb to 5.71 Mb and 4006 to 5179 genes. The G+C contents of the 20 *E. coli* genome were between 50.39% and 51.21%.

**Table 1:** Genome statistical information of the 20 *Escherichia coli* strains selected from NCBI.

Strain	Assembly.NO	Genome Size (Mb)	CDS	G+C (mol%)	Country	Isolation source	Host disease	Collection date
188B	GCA_014898615.1	5.13381	4096	51.21	Turkey	Blood	Sepsis	2018
188	GCA_014898635.1	5.1333	4006	51.11	Turkey	Blood	Sepsis	2018
2D	GCA_013425955.1	5.3633	4930	50.61	China	Blood	Bloodstream infection	2019
190693	GCA_011022275.1	5.15195	4714	50.60	UK	Blood	Bacteremia	2016
p11A	GCA_011330935.1	5.54584	5166	50.76	USA	Blood	Bacteremia	2016
p4A	GCA_011331215.1	5.2555	4861	50.70	USA	Blood	Bacteremia	2016
GZEC065	GCA_010092405.1	5.10792	4610	50.75	China	Blood	NA	2017
69	GCA_007107705.1	5.41794	5028	50.71	USA	Blood	Bloodstream infection	2014
95	GCA_007107905.1	5.06629	4662	50.72	USA	Blood	Bloodstream infection	2013
131	GCA_007108085.1	5.21412	4804	50.79	USA	Blood	Bloodstream infection	2012
963	GCA_007108305.1	5.25381	4756	50.70	USA	Blood	Bloodstream infection	2012
strain Z247	GCA_002142715.1	5.40007	4972	50.39	China	Blood	Bloodstream infection	2015
Z1002	GCA_002142675.1	5.62852	5152	50.52	China	Blood	Bloodstream infection	2014
SF-468	GCA_001280345.1	5.35864	5012	50.61	USA	Blood	Bloodstream infection	2010
CE10	GCA_000227625.1	5.37873	4911	50.58	USA	CSF	Neonatal meningitis	1970
RM-103-MS	GCA_014217095.1	5.70703	4353	50.50	Bangladesh	Feces	Healthy individual	2017
E686	GCA_011045295.1	5.16231	4516	50.60	Korea	Feces	NA	2019
2EC1	GCA_009905115.1	4.86859	4383	50.90	Singapore	Feces	Healthy individual	2019
SJ7	GCA_008761535.3	5.57204	5179	50.66	USA	Feces	Bloody diarrhea	2015
NCCP 15955	GCA_009873375.1	4.82062	4266	50.60	China	Feces	Diarrhea	2014

CDS, coding sequences; CSF, cerebrospinal fluid; NA, not available.

### Genomic Recombination Analysis

We used Roary to create a pan-genome and core-genome of 22 strains and got 2654 core genes (99%≤strains ≤ 100%) and 14,854 pan genes (0%≤strains ≤ 100%). Then we identified recombination with ClonalFrameML and got a pairwise single nucleotide polymorphisms distance matrix with snp-dists (**Supplementary 3**) from a FASTA sequence alignment after we masked the recombination regions. We found that the snp-dists between *E. coli* 413_bl and 413_csf was 0, which provided evidence that the two *E. coli* strains were highly homologous.

### Phylogenetic Analysis for *E. coli*

**Figure 4:**
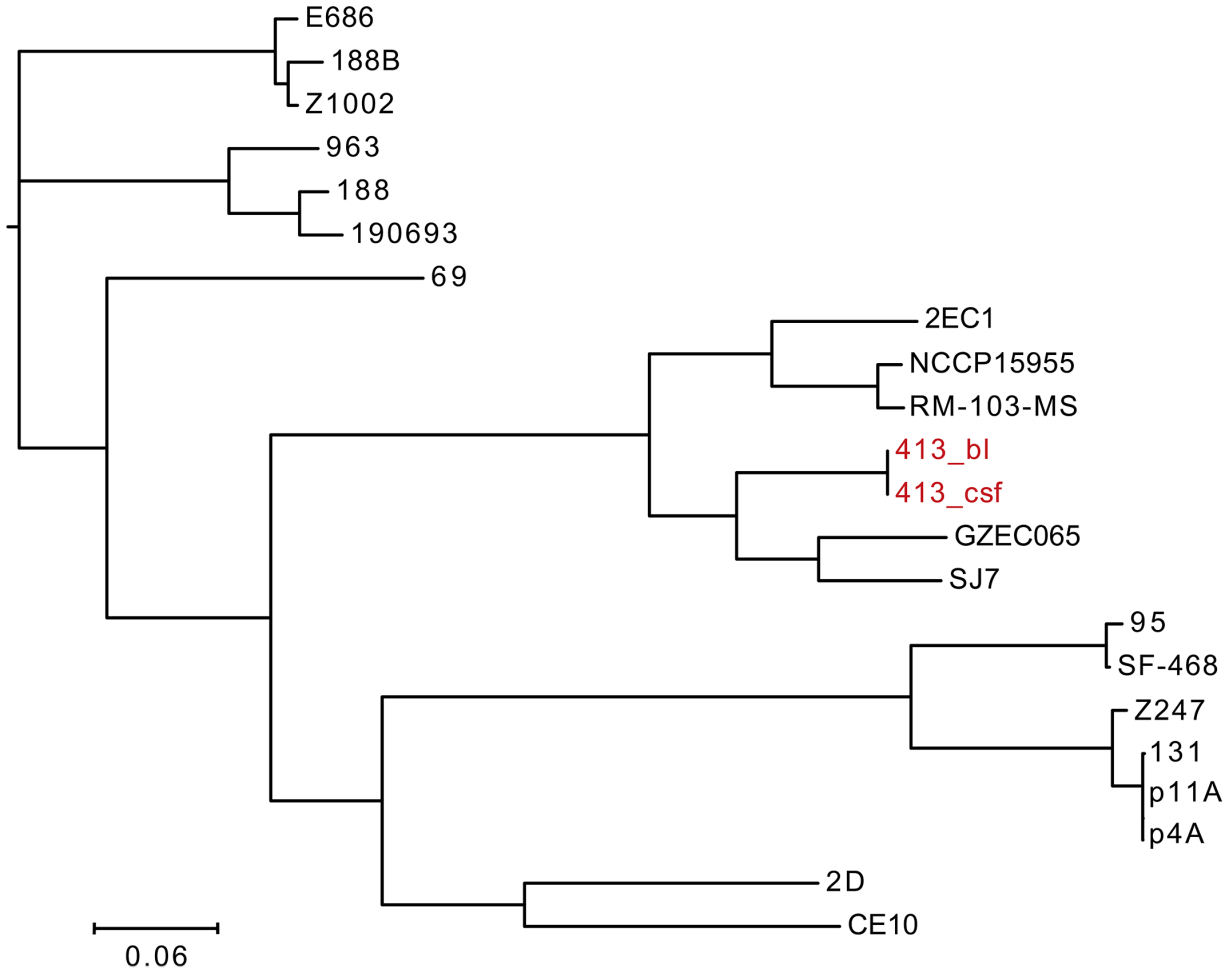
Phylogenetic tree constructed using whole genomes of 22 *E. coli* strains, including *E. coli* 413_bl strains isolated from bloodstream, *E. coli* 413_csf isolated from cerebrospinal fluid, and 20 *E. coli* strains screened from the National Center for Biotechnology Information.

We performed phylogenetic analysis for *E. coli* and conducted a phylogenetic tree based on the whole genome sequencing data of 22 *E. coli* strains **(** **Figure 4** **)**. It showed that *E. coli* 413_bl and 413_csf exist in the same clade with another five strains, including *E. coli* GZEC065, SJ7, 2EC1, NCCP15955, RM-103-MS, indicating that *E. coli* 413_bl and 413_csf have close genetic relationship with them. Combining the result that the snp-dists between *E. coli* 413_bl and 413_csf was 0, which further confirmed the 100% homologousness of them. *E. coli* GZEC065 was isolated from the blood of homo sapiens. *E. coli* SJ7, 2EC1, NCCP15955, and RM-103-MS were isolated from the feces of homo sapiens. Thus, we inferred that the *E. coli* 413_bl in our case may be from the intestine, and the *E. coli* 413_bl in the bloodstream entered the cerebrospinal fluid through the blood-brain barrier, causing intracranial infection. The only strain isolated from CSF, *E. coli* CE10, had a relatively far connection with *E. coli* 413_csf. We infer that that’s because *E. coli* CE10 was collected from newborn with bacterial meningitis in 1970s, which is ancient and not sourced from the intestine (Lu et al., 2011).

## Discussion

In this study, we identified *E. coli* from cerebrospinal fluid by mNGS under circumstance of initial traditional culture-negative, to assist the clinicians in detecting infectious pathogen rapidly. And the second cerebrospinal fluid culture reported *E. coli*, further indicating the precision of mNGS. It’s reported that the positive rate of cerebrospinal fluid culture in meningitis is only between 5.4% and 24.3% in developing countries like China (Li et al., 2014; Zhang et al., 2020). In addition, the prophylactic use of inappropriate broad-spectrum antibiotics reduces the sensitivity of pathogens detection by conventional culture, causing challenges in diagnosing the pathogens (Paul et al., 2010).

Accumulating evidence have proved that mNGS is an unbiased and comprehensive diagnostic strategy and is less disturbed by preliminary antibiotics exposure (Miao et al., 2018). A prospective multicenter study revealed that the positive rate of mNGS in cerebrospinal fluid infection was 57.0% (Xing et al., 2020). Moreover, it’s found that the patient—as in our study—with CSF WBC >300×10^6^/L, CSF protein > 500 mg/L, or glucose ratio ≤ 0.3 had a notably higher mNGS detection rate (Miao et al., 2018). Another retrospective observational study focused on CSF samples from 99 pediatric bacterial meningitis patients found that mNGS appeared with higher sensitivity (68.7%) than culture (55.6%), and the top four detected pathogens were *Streptococcus pneumoniae*, *Streptococcus Agalactiae*, *Staphylococcus aureus*, and *Escherichia coli* (Guo et al., 2019). In a word, it demonstrated that mNGS had preferable diagnosis efficiency to culture in CNS infection.

Postoperative CNS infection remains a severe question requiring instant attention in patients undergoing neurosurgery. A previous research in America found that 0.8% (14/1587) patients after cranial operations were complicated with intracranial infection, and the morbidity of bacterial meningitis was 0.3% (4/1587). In addition, this research observed no associations between cerebrospinal fluid leakage, diabetes mellitus, and CNS infection (McClelland and Hall, 2007). However, a retrospective study in China revealed that the risk of meningitis after major craniotomy was increased by the presence of diabetes mellitus and lumbar drainage (Chen et al., 2014). In our study, the patient had undergone surgery of craniopharyngioma resection by endoscopic endonasal transsphenoidal approach, with type 2 diabetes, cerebrospinal fluid leakage, and lumbar cistern drainage. No research reported the postoperative CNS infection associated risk factors of neurosurgical endoscopic endonasal transsphenoidal approach. Thus, it’s meaningful to further explore this topic to provide appropriate recommendations for clinicians around reducing the complications after neurosurgical procedures.

In addition to the identification of *E. coli* in the CSF, we also cultured *E. coli* in the bloodstream. To explore the homology of the two isolated *E. coli* strains, whole genome sequencing was performed. After phylogenetic analysis, we confirmed that the two *E. coli* strains posed 100% homologous—meaning they are the same strain, which helped clinicians inferring the source of *E. coli* in CSF. Because the patient occurred CNS infection post-surgery, we couldn’t exclude that the patient was infected during the surgery if without evidence. However, according to the results of whole genome sequencing, we concluded that the *E. coli* in CSF was originated from the bloodstream. In a way, it provides a reliable basis for the clinicians to clarify the causation to the patient. And the *E. coli* in bloodstream was carbapenem-resistant *via* drug susceptibility test, thus contributing to formulating a precise antibiotic treatment.

*E. coli* is a major pathogen that causes bloodstream infection. In Oxfordshire, UK, about half of *E. coli* bloodstream infections were associated with urinary tract infection and three quarters of bloodstream infection with *E. coli* were community-onset (Vihta et al., 2018). With the increase of antibiotic resistance, the scope of therapy choices is shrinking (Harris et al., 2018). Our study identified carbapenem-resistant *E. coli* from bloodstream infection in the hospital, which could be related to diarrhea. According to the statistics, carbapenem-resistant *Enterobacteriaceae* has been a major global public health problem (Xu et al., 2015). Patients with carbapenem-resistant *Enterobacteriaceae* infection usually have a history of prolonged exposure to health care facilities, which is consistent with long-term and diversified antibiotic consumption. Antibiotics available to treat carbapenem-resistant *Enterobacteriaceae* are limited, and combination therapy with multiple antibiotics has been shown to reduce mortality. In our study, anti-infective treatment was used for 20 days in combination with ceftazidime-avibactam and aztreonam, and the fever of the patient was relieved.

## Limitations

One pivotal limitation of our study was a lack of pathogen identification of the patient’s stool, meaning that our inferences about the relation of *E. coli* bloodstream infection and diarrhea need to be confirmed. Second, it’s important to administer early postoperative enteral nutrition to maintain the gastrointestinal homeostasis and the stability of gut microbiota. Third, we should strengthen the management of cerebrospinal fluid leakage in patients, trying to avoid the occurrence of it.

## Conclusion

In summary, our study demonstrated the usefulness of mNGS in improved identification of neurosurgical postoperative CNS infection. Actively tracing the infection source is of great significance to clinical diagnosis and treatment. In addition, carbapenem-resistant *E. coli* is a serious problem as the cause of bloodstream infection and CNS infection, and effective and adequate measures to prevent and control the present circumstance are urgent.

## Data Availability Statement

The data that support the findings of this study have been deposited into CNGB Sequence Archive (CNSA) of China National GeneBank DataBase (CNGBdb). The names of the repository/repositories and accession number(s) can be found below: https://db.cngb.org/, CNP0002005.

## Ethics Statement

The studies involving human participants were reviewed and approved by the ethical committee of the First Affiliated Hospital of Zhengzhou University (2019-KY-330). The patients/participants provided their written informed consent to participate in this study.

## Author Contributions

YL and MX designed the study. QS collected the data and performed the data analyses. JZ provided medication guidance. JW, LD, and ZS involved in patient management and provided clinical information. MX performed laboratory investigations. QS and JZ wrote the original manuscript. YL and MX revised and supervised the paper. All authors contributed to the article and approved the submitted version.

## Funding

This work was equally funded and supported by Chinese National Science and Technology Major Project 2018ZX10305410, Henan Province Science and Technology Research Project grant 192102310054 and 212102310321, Fund of the First Affiliated Hospital of Zhengzhou University 2018020136.

## Conflict of Interest

The authors declare that the research was conducted in the absence of any commercial or financial relationships that could be construed as a potential conflict of interest.

## Publisher’s Note

All claims expressed in this article are solely those of the authors and do not necessarily represent those of their affiliated organizations, or those of the publisher, the editors and the reviewers. Any product that may be evaluated in this article, or claim that may be made by its manufacturer, is not guaranteed or endorsed by the publisher.
